# Nanoscopic Characterisation of Individual Endogenous Protein Aggregates in Human Neuronal Cells

**DOI:** 10.1002/cbic.201800209

**Published:** 2018-09-11

**Authors:** Daniel R. Whiten, Yukun Zuo, Laura Calo, Minee‐Liane Choi, Suman De, Patrick Flagmeier, David C. Wirthensohn, Franziska Kundel, Rohan T. Ranasinghe, Santiago E. Sanchez, Dilan Athauda, Steven F. Lee, Christopher M. Dobson, Sonia Gandhi, Maria‐Grazia Spillantini, David Klenerman, Mathew H. Horrocks

**Affiliations:** ^1^ Department of Chemistry University of Cambridge Lensfield Road Cambridge CB2 1EW UK; ^2^ UK Dementia Research Institute University of Cambridge Cambridge CB2 0XY UK; ^3^ Department of Clinical Neurosciences University of Cambridge Hills Road Cambridge CB2 0AH UK; ^4^ UCL Institute of Neurology Queen Square London WC1N 3BG UK; ^5^ Present addresses: EaStCHEM School of Chemistry University of Edinburgh David Brewster Road Edinburgh EH9 3FJ UK; ^6^ UK Dementia Research Institute University of Edinburgh Edinburgh UK

**Keywords:** amyloid formation, aptamers, DNA PAINT, induced pluripotent stem cells, neurodegenerative disorders, alpha-synuclein

## Abstract

The aberrant misfolding and subsequent conversion of monomeric protein into amyloid aggregates characterises many neurodegenerative disorders, including Parkinson's and Alzheimer's diseases. These aggregates are highly heterogeneous in structure, generally of low abundance and typically smaller than the diffraction limit of light (≈250 nm). To overcome the challenges these characteristics pose to the study of endogenous aggregates formed in cells, we have developed a method to characterise them at the nanometre scale without the need for a conjugated fluorophore. Using a combination of DNA PAINT and an amyloid‐specific aptamer, we demonstrate that this technique is able to detect and super‐resolve a range of aggregated species, including those formed by α‐synuclein and amyloid‐β. Additionally, this method enables endogenous protein aggregates within cells to be characterised. We found that neuronal cells derived from patients with Parkinson's disease contain a larger number of protein aggregates than those from healthy controls.

Protein misfolding and aggregation is closely associated with the development of many neurodegenerative disorders, such as Alzheimer's disease (AD) and Parkinson's disease (PD).^1^ In AD, the protein tau is deposited in intracellular inclusions,^2^ while the amyloid beta (Aβ) peptide is in extracellular plaques. Similarly, in PD, aggregates of the protein α‐synuclein (αS) are found in Lewy bodies^3^ within neuronal cells. These proteins are often heavily post‐translationally modified, for example, αS undergoes phosphorylation, nitration and truncation,^4^, ^5^, ^6^ this makes it important to be able to characterise the real endogenous aggregates formed in cells, as these can differ from those formed by unmodified proteins.

Soluble nanometre‐sized protein oligomers have been identified as the major cytotoxic species in AD and PD,^7^, ^8^, ^9^, ^10^ but the study of such species has remained challenging, as they tend to be low in abundance and adopt a wide range of heterogeneous structures. To overcome this problem, we have developed an array of single‐molecule techniques^11^, ^12^, ^13^, ^14^ to observe oligomeric species individually, and have applied them to characterise the aggregation pathway of several disease‐related proteins in vitro. In many such methodologies, the protein of interest needs to be tagged with either an organic fluorophore or a fluorescent protein. This is very challenging for in vivo or in cell imaging, and in some cases the label can have an adverse effect on the behaviour of the protein.^15^ Alternatively, dyes such as thioflavin‐T/S (ThT/S) or the pentameric form of formyl thiophene acetic acid (pFTAA), whose fluorescence in each case is enhanced upon binding to amyloid structures, can be used to detect protein aggregates. We have recently used such dyes in combination with total internal reflection fluorescence (TIRF) microscopy to image individual aggregates in human cerebrospinal fluid (CSF) in a diffraction‐limited manner.^16^ Such dyes, however, bind to other cellular components, thus limiting their versatility,^17^ and might not be sensitive to the smaller oligomers that, in addition to being major therapeutic targets, could also be biomarkers for neurodegeneration.^16^, ^18^ Furthermore, conventional far‐field microscopy techniques face a limit in the resolving capability imposed by the optical diffraction barrier. As many subcellular structures are known to be affected by toxic protein aggregates,^19^, ^20^ it is important to define the morphology and location of aggregates in the cellular milieu in order to understand the interplay between protein aggregation and the loss of cellular homeostasis.

We have used an aptamer previously reported to recognise oligomers and fibrils formed from αS and Aβ^21^ to enable the sensitive and specific visualisation of protein aggregates at the nanoscale. Aptamers are single‐stranded oligonucleotides developed to have high affinity and specificity and can be made for almost any molecule or structure.^22^, ^23^ The advent of super‐resolution (SR) microscopy^24^ has improved optical methods. Recently, an SR method, referred to as DNA PAINT (point accumulation in nanoscale topography), has been developed.^25^, ^26^ The technique uses short complementary strands of DNA: a “docking” strand is conjugated to an antibody or a protein of interest, whilst its complementary “imaging” strand is labelled with an organic fluorophore. We extended the aptamer sequence with a docking strand sequence (Figure 1 A, Table S1 in the Supporting Information) to generate SR images of protein aggregates (Figure 1 D); we refer to this method as aptamer DNA PAINT (ADPAINT). Repeated transient binding of the imaging strands to the docking strand (Figure 1 A–C) allows the labelled biomolecule to be spatially localised and enables the reconstruction of an SR image. Additional burst montages are shown in Figure S2 and provide a more complete view of the variation in fluorescent bursts caused by the stochastic binding of the imaging strands to the docking strand. DNA PAINT works with both TIRF microscopy and, more recently, spinning‐disk confocal microscopy.^27^ Examples of both αS and Aβ oligomers and fibrils imaged by using ADPAINT are shown in Figure 1 D (full fields of view are shown in Figures S3 and S4). A control experiment on just the imaging strand (without aptamer) showed little nonspecific binding of the imaging strands to the aggregates (Figure S5). The aptamer is specific to the conformation of the aggregates, so these can be detected even amongst an excess of monomers. Furthermore, for primary and secondary antibodies, the size of a probe can add a linkage error of 15 nm,^28^ whereas the small size of aptamers enables them to bind at a higher density and at closer proximity to their epitopes; this leads to a higher imaging resolution, as has also been shown with DNA PAINT and non‐antibody binding proteins such as affimers.^29^ Typically, we achieve a localisation precision of ≈10 nm and a resolution of ≈25 nm (Table S2), with a limit of detection of ≈30 pm of aggregates (see the Supporting Information). This enables us to quantify the oligomers formed during physiologically relevant aggregation reactions. Each image was acquired over 200 s; however, as PAINT‐based techniques are not limited by photobleaching,^30^ this time can be lengthened to localise a greater number of binding events in order to obtain a higher‐resolution image of the protein or the cellular structure of interest.

**Figure 1:**
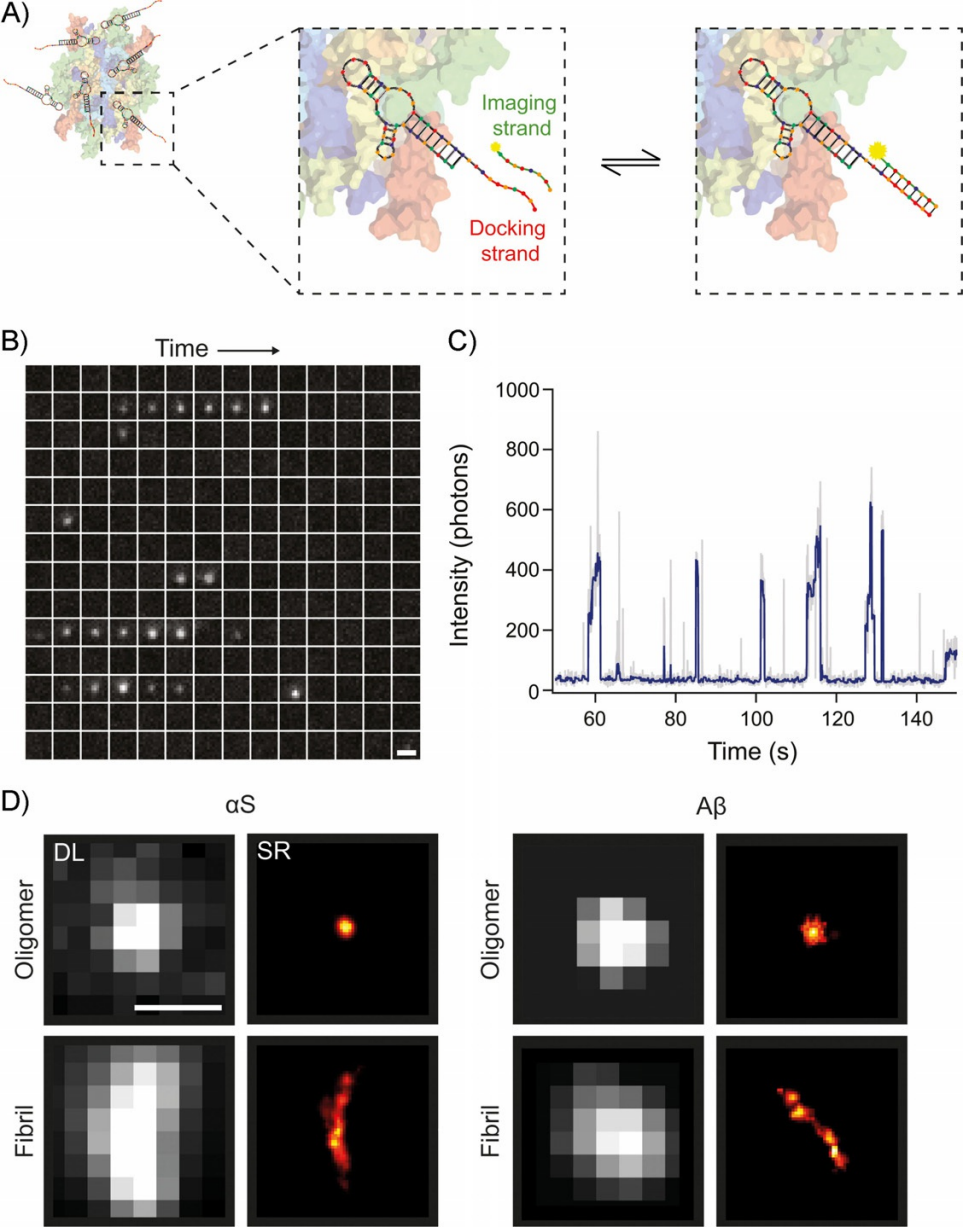
The concept of ADPAINT. A) Schematic representation of ADPAINT showing an aggregate bound by multiple aptamers. The DNA docking strand on the aptamer is transiently bound by the complementary imaging strand to generate a SR image. B) Example time montage of an oligomer undergoing ADPAINT. Each sub‐image is separated by 0.5 s, moving through time from left to right then top to bottom; scale bar: 1 μm. C) Intensity profile of the oligomer in (B). Each intensity burst represents the binding of the imaging strand to the aptamer. Grey: raw intensity profile, blue: using a Chung–Kennedy filter^31^ with a window of five frames applied. D) Examples of diffraction‐limited (DL, using thioflavin‐T) and super‐resolved (using ADPAINT) images of an αS and Aβ oligomer and fibril. Scale bar: 500 nm.

To assess the ability of ADPAINT to study the heterogeneity of complex aggregation mixtures, a solution of monomeric αS was incubated under conditions previously found to result in its aggregation.^7^, ^11^ At early time points in the reaction, only a few aggregates were detected, and these were predominantly small (<400 nm in length for the first 6 h) and rounded; this is consistent with the expected appearance of oligomers (Figure 2 A). After 10 h, fibrils were detected. To visualise the distribution of binding sites within each aggregate, we colour‐coded the localisations according to their local (typically within 40–50 nm) molecular density. The resulting images show maps of the local molecular density of individual aptamer binding sites, which reveal a highly non‐uniform distribution, particularly in the later aggregates (right panel in each case). This shows that the aggregate structure is not homogeneous, but instead varies at the nanoscale, a finding that is made possible by this method. Further analysis of the ADPAINT images showed that the number of aggregates increased over time; this is consistent with the high aggregation propensity of αS (Figure 2 B). Unlike antibodies in which stoichiometric labelling can be challenging, each aptamer is labelled with a single DNA docking strand, thus allowing quantitative imaging. We took advantage of this by quantifying the number of localisations per aggregate and found that this increased over time (Figures 2 C and S6). Additionally, the aggregates also became larger, as indicated by the increase in their mean length (Figures 2 D and S7).

**Figure 2:**
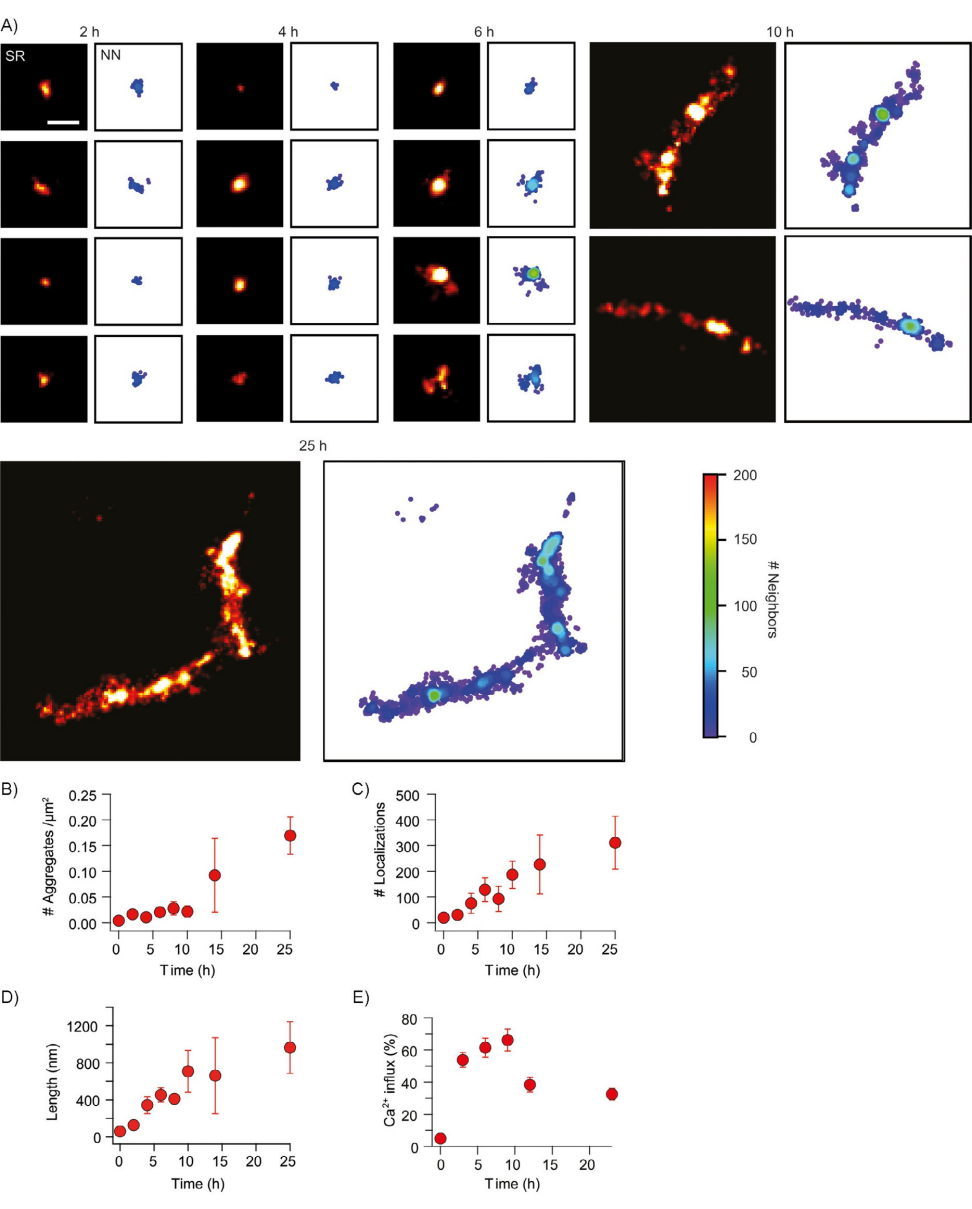
ADPAINT enables the imaging of a range of species formed during the aggregation of αS. A) Example aggregates are shown in SR on the left, with their corresponding nearest neighbour (NN) plots shown on the right, highlighting hotspots of localisation density. Scale bar: 200 nm. B) The number of aggregates increases over time, and C) the number of localisations also increases as the species get larger; this is shown by D) the mean length increases. Data shown are mean±SD of three independent aggregation reactions. E) The percentage of liposomes permeabilised upon addition of aggregates from the different time‐points (mean±SD over 16 fields of view (69×69 μm)).

The permeabilisation of membranes has been suggested to be the most ubiquitous toxic mechanism associated with protein aggregates.^31^, ^32^, ^33^, ^34^, ^35^ We have developed a method to characterise the ability of protein aggregates to permeabilise lipid membranes^8^ (details are given in the Supporting Information) and applied it in this study. We found that the earlier aggregates caused a higher level of influx than those present at later stages of the aggregation process (those around 600 nm in length; Figure 2 E). Additionally, the binding of the aptamer to the aggregates did not inhibit their ability to permeabilise the lipid membranes, and the aptamer itself displayed no propensity to alter these membranes (Figure S8). Thus, it appears likely that ADPAINT can be applied to characterise the structures of the pathological aggregates without altering the functional states of the protein or the cell membrane.

We next used ADPAINT to investigate aggregate formation in a cellular model of PD. Mis‐sense mutations^13^, ^36^, ^37^, ^38^, ^39^, ^40^ and duplications or triplications of the *SNCA* gene, which encodes αS, lead to autosomal dominant early onset PD.^41^, ^42^ It has previously been shown that the formation of αS oligomers in vitro is concentration dependent,^12^ and ADPAINT now enables us to determine whether this dependence is reflected in cellular models that overexpress αS. We used induced pluripotent stem cells (iPSCs) from a PD patient with a triplication of the *SNCA* gene and from a healthy control unaffected by the disease to generate cortical neurons. Although SR methods have been used to image fibrils in cells, these are typically exogenously added aggregates generated from fluorophore‐labelled protein.^28^, ^43^, ^44^, ^45^, ^46^, ^47^ This is the first case in which a specific probe for aggregates has been used, and it enables the SR imaging of unlabelled, endogenous aberrant protein complexes. These were imaged in fixed, permeabilised cells by using both ADPAINT at the SR level and in a diffraction‐limited manner by using pFTAA, a green dye that recognises β‐sheet structures and becomes fluorescent upon binding to protein aggregates.^48^, ^49^ To image at a greater depth into the cells, the illumination was changed from TIRF to oblique‐angle epifluorescence. Figure 3 shows examples of human iPSC‐derived neurons with and without the *SNCA* triplication after they have been plated and stained with pFTAA (further examples are shown in Figure S9). PFTAA not only binds to the aggregates, which appear as brighter spots, but also interacts with cellular organelles and membranes, thereby preventing the aggregates from being identified or their precise location within the cell from being determined. Unlike pFTAA, the aptamer has a high specificity, and only small clusters of binding events are detected within the cytosol. Due to the background fluorescence being higher in oblique‐angle epifluorescence than in TIRF, the resolution we achieved within cells was lower than the resolution achieved for the aggregates formed in vitro (Table S2). Quantification of these images shows that there are significantly (*p*<0.0001) more aggregates in the cells derived from the individual carrying a triplication of the *SNCA* locus compared to iPSC‐derived neurons from the healthy control. We found that the species detected in these experiments resemble those formed early on in the in vitro aggregation pathway (0–2 h) shown in Figure 2, having ≈45 localisations per aggregate (Figure 3 C), and being <150 nm in length (Figure 3 D). Furthermore, the aggregates detected in the cells having the *SNCA* triplication locus give rise to significantly more localisations (Figure 3 C) and are larger (Figure 3 D) than those in the healthy control cells. Given the likelihood of toxicity arising directly from these aggregates, this observation could help explain the neuronal cell death associated with PD.

**Figure 3:**
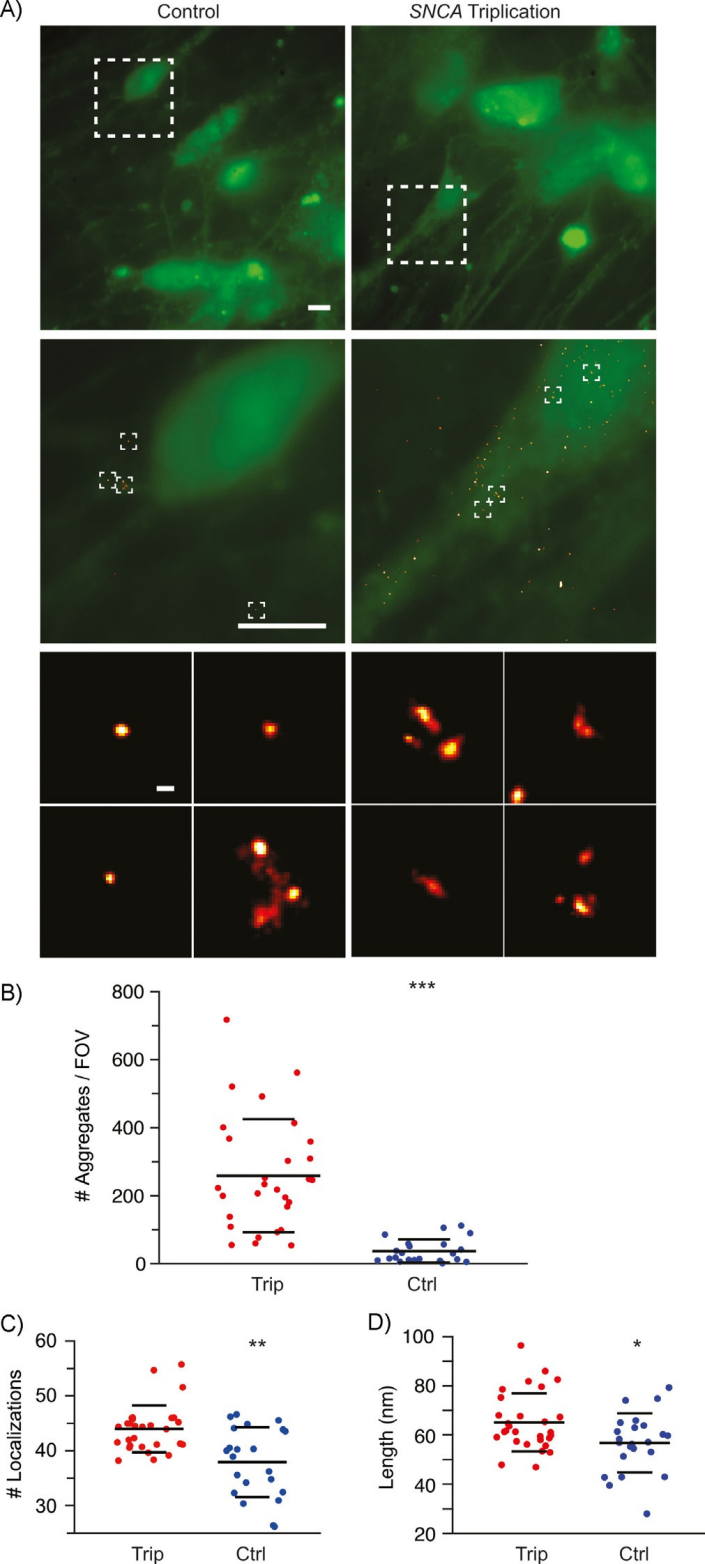
ADPAINT in iPSCs. A) iPSCs from a PD patient with a triplication of the *SNCA* gene and from a healthy control. Protein aggregates were imaged by using pFTAA (green) or ADPAINT (red). Scale bars: 5 μm (top) and 100 nm (middle). Compared to control cells, *SNCA* triplication cells show B) significantly more aggregates and increases in C) the number of localisations and D) the average length of the aggregates. The data shown are means±SD of at least 27 fields of view. * *p*<0.05, ** *p*<0.001, *** *p*<0.0001; analysed by t‐test.

One of the significant advantages of ADPAINT is the ability of the aptamer to selectively bind to protein aggregates but not the excess of monomeric protein that is present in cells. As a comparison, we used the commercially available MJF14‐6‐4‐2 filament antibody, which detects an epitope that is only accessible in aggregates but not in the monomeric protein,^50^ and an Alexa Fluor 405–labelled aptamer to detect dual‐labelled aggregates of αS added to iPSC‐derived neurons (Figure S10). In the case of the MJF14‐6‐4‐2 antibody, there was nonspecific staining of regions of the cell that did not contain aggregated αS, whereas the aptamer only detected dual‐labelled aggregates. Furthermore, the larger size of antibodies can add a further 10–15 nm between the target and the labelled probe.^28^ When used with DNA PAINT, the same MJF14‐6‐4‐2 antibody (Figure S11), was unable to resolve individual aggregates, but instead there was diffuse staining in both the *SNCA* triplication and control cells.

In conclusion, we have developed an SR method to characterise the toxic species formed during neurodegenerative diseases. We have used ADPAINT to characterise both in vitro aggregates αS and Aβ, as well as endogenous unlabelled oligomers formed in patient‐derived neurons. Interestingly, we found that the aggregates formed early on in the in vitro aggregation closely resemble the morphology of those found within human iPSC‐derived cortical neurons and that these appear to be most responsible for disrupting lipid membranes. Although only one stage in the lifetime of the iPSC‐derived cortical neurons was imaged, this method can also be used to determine how such species develop as cells age, potentially yielding further insights into the progression of neurodegenerative diseases.

## Conflict of interest

*The authors declare no conflict of interest*.

## Supporting information

As a service to our authors and readers, this journal provides supporting information supplied by the authors. Such materials are peer reviewed and may be re‐organized for online delivery, but are not copy‐edited or typeset. Technical support issues arising from supporting information (other than missing files) should be addressed to the authors.

Supplementary
