# Supplementary material for: Nanoscopic Characterisation of Individual Endogenous Protein Aggregates in Human Neuronal Cells
Source: Chembiochem. 2018 Sep 11;19(19):2033–8. doi: 10.1002/cbic.201800209 (PMC6220870; doi:10.1002/cbic.201800209)
Supplement: Supplementary file 1 — Supplementary [file CBIC-19-2033-s001.pdf]

CHEMBIOCHEM

Supporting Information

Nanoscopic Characterisation of Individual Endogenous Protein Aggregates in Human Neuronal Cells

Daniel R. Whiten+,[a] Yukun Zuo+,[a] Laura Calo,[c] Minee-Liane Choi,[d] Suman De,[a] Patrick Flagmeier,[a] David C. Wirthensohn,[a] Franziska Kundel,[a] Rohan T. Ranasinghe,[a] Santiago E. Sanchez,[a] Dilan Athauda,[d] Steven F. Lee,[a] Christopher M. Dobson,[a] Sonia Gandhi,[d] Maria-Grazia Spillantini,[c] David Klenerman,*[a, b] and Mathew H. Horrocks*[a, e, f]

cbic_201800209_sm_miscellaneous_information.pdf

Table of Contents

Experimental Procedures 2
Data analysis 4
Limit of detection 5

Supporting Information Tables:
Supporting Information Tables 1: Sequences of DNA constructs used in this work. 5
Supporting Information Tables 2: Resolution as determined by Fourier ring correlation. 5

Supporting Information Figures:
Supporting Information Figure 1: Concentration calibration curve 6
Supporting Information Figure 2: Example burst montages. 7
Supporting Information Figure 3: Example full field of view images of αS aggregates 8
Supporting Information Figure 4: Example full field of view images of Aβ aggregates 9
Supporting Information Figure 5: Non-specific binding of imaging strand to αS aggregates 9
Supporting Information Figure 6: Timecourse - number of localizations histogram 10
Supporting Information Figure 7: Timecourse - lengths histogram 11
Supporting Information Figure 8: Membrane permeabilization assay 12
Supporting Information Figure 9: Example images from triplication and control iPSC-derived cortical neurons (uploaded as Supporting data) 12
Supporting Information Figure 10: Comparison between antibody and aptamer staining 13
Supporting Information Figure 11: Comparison between DNA PAINT and ADPAINT 13
Supporting Information Figure 12: Demonstration of the skeletonization in the data analysis 14

Supporting References 14

Author Contributions 14

Experimental Procedures

Preparation of coverslides

Glass coverslips (0.13 mm thickness, round, 50 mm diameter) were cleaned using an argon plasma (PDC-002, Harrick Plasma) for 1 h. Then, a multiwell chamber coverslip (CultureWell CWCS-50R-1.0, 50 channels) was cut in half and affixed to the glass coverslip. The slide was incubated with aspartic acid (Sigma, 1 mg/mL) for 1 h and washed once with Tris buffer (0.02 μm filtered, Anotop25, Whatman); 5.0 mM tris(hydroxymethyl)aminomethane (Tris), 1 mM EDTA, 10 mM MgCl2, pH 8). Samples were incubated on the slide for 5 min, before the liquid was removed and replaced with Tris buffer containing aptamer, imaging strand and/or ThT/pFTAA. The slides were then transferred to the microscope stage and coupled to the objective using refractive index-matched immersion oil (refractive index n = 1.518, Olympus, UK). If imaging for longer than 2 h, another clean coverslip was then layered on top of the multiwell chamber to prevent evaporation.

Preparation of aptamer and fluorophores

All DNA strands were diluted in Tris buffer. The docking and imaging strand sequences were the same as those used previously[1]. The docking strand-conjugated aptamer (ATDBio, sequence provided in Table S10) was used at a final concentration of 100 nM. The DNA imaging strand (ATDBio) was also dissolved in Tris buffer and used at a final concentration of 1 nM. Thioflavin T (ThT) (Sigma) was dissolved in absolute ethanol (99%) and then diluted in Tris buffer and filtered (0.02 μm filtered, Anotop25, Whatman). The exact concentration was determined by Nanodrop ($\varepsilon_{421\ nm}$ = 36 000 M^{-1} cm^{-1}). The ThT solution was further diluted to 5 μM in Tris buffer and mixed with the abovementioned two DNA solutions for sequential single aggregate visualization by enhancement (SAVE) imaging[2]. The final concentration of ethanol was < 5%.

Protein purification and aggregation

Wild type αS was purified from E. coli as previously described[3]. It was stored at −80 °C. When thawed, the solution was centrifuged at 20,000 x g for 2 h to remove insoluble pre-aggregated seeds. The αS solution was divided into three samples, each of which was diluted to 70 μM in Tris buffer. The final volume of the sample was around 200 μL. The solution was then incubated at 37 °C with 200

rpm orbital shaking. Each reaction was supplemented with sodium azide (0.01%, m/m) to prevent bacterial growth. Aliquots (10 µL) were taken at the indicated times and flash frozen before use.
Synthetic Aβ42 peptide was produced by Anaspec (USA). As previously described, the peptide was dissolved in 10 mM NaOH, purified by high-performance liquid chromatography using a BioSep-SEC-S2000 column (Phenomenex) and flash frozen before use. Aβ42 was aggregated at a final concentration of 2 µM at 37 °C with 200 rpm orbital shaking.

Preparation of Human pluripotent stem cells

Fibroblasts obtained from skin biopsies from a healthy individual were reprogrammed into iPSCs at the Human iPS core facility, LRM (University of Cambridge, UK)[4]. PD patient iPSCs harbouring a triplication of the SNCA gene encoding αS, were reprogrammed from skin biopsies fibroblasts by the Coriell Biorepository (Camden, USA).
IPSCs were grown in TeSR-E8 (STEMCELL technologies, UK) in feeder-free conditions. Confluent iPSCs were re-plated and grown in neuronal induction medium (NIM, Gibco, Life technologies, UK) to obtain neural stem cells (NSCs; day 1). After NSCs expansion in NIM, cells were plated onto coated glass coverslips (VWR International, Belgium) and terminal differentiation promoted by exposure to 10µM N-[N-(3,5-difluorophenacetyl)-l-alanyl]-S-phenylglycine t-butyl ester; DAPT) for 10 days in neuronal differentiation medium (NDM) to generate a mixed population of mature forebrain neurons following a method adapted from Kirkeby et al., 2012[5]. Neurons were maintained up to day 50 in NDM.

Preparation of DNA labeled MJF14-6-4-2 antibody

Lyophilized thiolated docking strand received from the supplier (ATDBio, Southampton, UK) was dissolved in 100 µL 1× PBS (pH 8.5, containing 50 mM DTT, dithiothreitol) and incubated for 30 minutes to reduce any residual disulfides. This solution was then diluted with water (400 µL) and extracted with ethyl acetate (3 × 500 µL). The DTT-treated oligonucleotide was then desalted using a NAP-5 column (GE Healthcare, Cat. No. 17085301), its concentration calculated from A_{260} (Nanodrop 2000, ThermoFisher Scientific) and aliquots stored at −20 °C.

The antibody was covalently coupled to thiolated docking strand by conjugate addition as described by Schnitzbauer *et al.*[1], with only minor modifications to the protocol. Anti-Alpha-synuclein filament antibody (MJF14-6-4-2), BSA and Azide free (abcam, Cambridge, UK, Cat. No. ab214033, Lot. No. GR310602-4) was first concentrated to ~12 µM (~1.7 mg/mL) in 1× PBS using Amicon spin filter (100 kDa MWCO) and then activated for reaction with thiolated docking strand by adding 0.1 volumes of 1.2 mM maleimide-PEG$_2$-succinimidyl ester crosslinker (Sigma Aldrich, Cat. No. 746223, in 1× PBS/6% *N,N*-dimethylformamide). This solution was kept at 4 ºC for 90 minutes, then excess crosslinker removed using a Zeba Spin Desalting Column, 7K MWCO (ThermoFisher, Cat. No. 89882). The antibody concentration was then checked by A_{280}, a 10-fold molar excess of thiolated docking strand added, and the solution incubated at 4 ºC overnight. After the reaction, excess thiolated DNA was removed using Amicon spin filter (100 kDa MWCO) and the concentration of antibody and degree of labeling (~3 docking strands per antibody) measured using A_{260} and A_{280}.

Super resolution imaging of cells

To prepare cells for imaging they were first washed with phosphate buffered saline (PBS) before being fixed with paraformaldehyde (4% w/v) for 15 min at room temperature. The cells were rinsed three times with PBS before being incubated for 1 h at room temperature in PBS containing 0.5% triton X-100 with 10% goat serum. The aptamer and imaging strand was then diluted to 100 nM and 5 nM respectively in PBS + 10% goat serum. The cells were incubated with the aptamer solution overnight at 4°C before imaging. Near-TIRF illumination was used to illuminate the cells.

For the imaging with the MJF14-6-4-2 antibody, the fixed and permeabilised cells were incubated with the antibody at a concentration of 1 µg/ml in PBS + 10% goat serum. The cells were washed three times with PBS before being imaged with 5 nM imaging strand.

Single vesicle assay

Vesicles are prepared by mixing 1-palmitoyl-2-oleoyl-sn-glycero-3-phosphocholine 16:0-18:1 PC (Avanti Polar Lipids) and 1-oleoyl-2-[12-biotinyl(aminododecanoyl)]-sn-glycero-3-phosphocholine 18:1-12:0 Biotin PC (Avanti Polar Lipids) at 100:1 ratio. The solvent was then removed under vacuum in a desiccator overnight and the lipids were then rehydrated in HEPES buffer (pH 6.5) with 100 µM Cal-520 (Stratech). Five freeze-and-thaw cycles were performed using dry ice and a water bath, followed by 10 times extrusion (Avanti Polar Lipids) with a membrane of 200 nm cut off to improve the homogeneity of the vesicle size and lamellarity distribution. To separate non-incorporated dye molecules from the vesicles, size-exclusion chromatography was performed. Then the purified vesicles tethered to PLL-PEG coated borosilicate glass coverslides using a biotin-neutravidin-biotin linkage and incubated in Ca^{2+} containing (1.26 Mm) Leibovitz's L-15 solution (Thermo-fischer). The frame-seal incubation chambers affixed (Biorad) coverslides were placed on the instrument described below. Thereafter the sample, diluted to a concentration of twice the targeted value, was added to the coverslide and incubated for ~10 min and same field of views are imaged. Next, ionomycin (Cambridge Bioscience Ltd), an ionophore for Ca^{2+}, was added and incubated for 5 min and subsequently images of Ca^{2+} saturated single vesicles in the same fields of view were acquired once more. Importantly, we made sure that the coverslides were not moved during the experiment so that exact same vesicles can be

imaged in different conditions, which can be analyzed to calculate sample induced Ca^{2+} influx quantitatively. For each field of view 50 images were taken with an exposure time of 50 ms each. The recorded images were analyzed using ImageJ (National institute of Health) to determine the fluorescence intensity of each spot under the three different conditions, namely blank (F_{blank}), in the presence of an aggregation mixture (F_{sample}), and after the addition of ionomycin ($F_{Ionomycin}$). Spot detection was implemented using 'find maxima' and intensity of the spots are calculated by considering 5 pixel diameter by centering brightest pixel of the spot. Then the particular sample induced relative Ca^{2+} influx into a vesicle was calculated using the following equation:

$$Ca^{2+}\text{influx} = \frac{F_{sample} - F_{blank}}{F_{Ionomycin} - F_{blank}} \quad (1)$$

The average degree and standard deviation of Ca^{2+} influx was calculated by averaging the Ca^{2+} influx into individual vesicle from 16 field of views.

Instrumentation

A home-built total internal reflection fluorescence (TIRF) microscope was used for imaging. The output from two lasers operating at 405 nm (Oxxius Laser-Boxx, Oxxius) and 561 nm (Cobalt Jive, Cobalt) were aligned to the optical axis of a 1.49 N.A., 60x TIRF objective (UPLSAPO, 60XO TIRF, Olympus) mounted on an inverted Ti-E Eclipse microscope (Nikon, Japan). The microscope was fitted with a perfect focus system (PFS) which auto-corrects the z-stage drift during a prolonged period of imaging. The laser power was attenuated by neutral density filters before the beam was passed through a quarter-wave plate (to circularly polarize the laser beam), a beam expander and their respective excitation filters (FF01-417/60-25 for 405 nm and FF01-561/14-25 for 561 nm, Semrock). The lasers were combined by a dichroic mirror (FF458-Di02-25x36, Semrock) and passed through the back port of the microscope and focussed in the sample by the objective. The laser power at the objective was 1.13 mW and 10.07 mW for the 405 nm and 561 nm lasers respectively. Fluorescence was collected by the objective and separated from the excitation light by a dichroic mirror (Di01-R405/488/561/635, Semrock), and passed through appropriate filters (BLP01-488R-25 for ThT and LP02-568RS-25 for Cy3B, Semrock). The fluorescence was then passed through a 2.5x beam expander and recorded on an EMCCD camera (Evolve 512, Photometrics) operating in frame transfer mode (EMGain of 11.5 e^{-1}/ADU and 250 ADU/photon). Each pixel corresponded to a length of 131.5 nm. Images were taken in a grid by an automated script (Micro-Manager) to prevent user bias. Exposure times were kept constant at 50 ms; to ensure that each bound aptamer was localized only once on average, 4000 frames were acquired for SR images (see Supporting Information). 100 frames were acquired for diffraction limited images.

Data Analysis

The positions of the transiently immobilised imaging strands within each frame were determined using the PeakFit plugin (an imageJ/Fiji plugin of the GDSC Single Molecule Light Microscopy package (http://www.sussex.ac.uk/gdsc/intranet/microscopy/imagej/gdsc_plugins) for imageJ (ref. [6]) using a typical 'signal strength' threshold of 30 and a precision threshold of 20 nm. The localizations were sorted into clusters using the DBSCAN algorithm in Python 2.7 (sklearn v0.18.1) using epsilon = 3 pixels and a minimum points threshold of 10 to remove spurious localisations. For each localization in the cluster, the shortest distance to the neighboring localizations was calculated and defined as the nearest neighbor (NN) distance. Molecular positions were plotted and color-coded on basis of the local density, defined as the number of molecules within a radius of 5 times the mean nearest neighbor distance of all molecules in that cluster. The number of clusters, localisations per cluster and nearest neighbor analyses were determined using custom scripts for Igor Pro (Wavemetrics). The resolution was determined by plotting a Fourier ring correlation curve (GDSC SMLM package)[7] for each image and determining the spatial frequency at which the curve drops below 1/7[8]. The resolutions for each of the datasets are provided in Supporting Information Tables 2. For cell imaging, localizations from the nuclei were not included for further analysis.

In order to ensure that the majority of aptamers were imaged at least once, but not multiple times, the imaging strand concentration was used at <10 nM. Under identical conditions, individual surface-immobilized biotinylated docking strands (via a biotin-streptavidin sandwich) were also imaged using DNA PAINT. This gave rise to a localization rate of 0.0069 binding events s^{-1} per docking strand, and so the ADPAINT images were captured over 200 s. At this length of time, 72% of the identified docking strands had one binding event, and 91% less than two binding events.

The lengths and eccentricities of the aggregates were calculated to structurally characterize the super-resolved aggregates. Using a custom script in Python 2.7, the eccentricity of each cluster identified by DBSCAN was determined, before the clusters were skeletonized using SciPy v0.18.1 (Supporting Information Figure 11). To limit the branching of the cluster skeletons the resulting image was blurred with a Gaussian filter (sigma = 2) before the clusters were skeletonized again. The blurring did not cause a loss of resolution in comparison to the original super resolution image. The lengths of the skeletons were then calculated for each cluster.

Limit of detection

To determine the limit of detection of ADPAINT, a sample of the t = 6 h αS aggregation was diluted to a series of concentrations. The absolute concentration of aggregates was determined by noting that at this time, 6.7% of the monomer was incorporated into the fibrils (determined by comparison with the number of species detected at the end of the aggregation), and that the mean fibril length at this time-point was 142.6 nm, which corresponds to 303 monomer units (one monomer for every 0.47 nm of fibril length[9]). A four-parameter logistic regression was used to fit the data, enabling future determination of absolute concentrations (Supporting Information Figure 1).

The limit of blank (LoB) is the highest apparent number of counts expected to be found when replicates of a sample containing no analyte are detected, and is given by the expression[10]:

$$LoB = \text{mean blank} + 1.645 \times (\text{SD of blank}) \quad (2)$$

The limit of detection (LoD) was determined by utilizing both the measured LoB and test replicates of a sample known to contain a low concentration of analyte, and is defined as LoD:

$$LoD = LoB + 1.645 \times (\text{SD of low concentration sample}) \quad (3)$$

The LoB was determined to be 2.39 x 10^{-3} aggregates/μm^2, and the LoD to be 6.59 x 10^{-3} aggregates/μm^2. Therefore, this method can accurately detect oligomers at concentrations as low as ~30 pM.

Supporting Information Tables

Supporting Information Table 1: Sequences of DNA constructs used in this work.

DNA strand name	Sequence
Aptamer	GCCTGTGGTGTTGGGGCGGGTGCG
Aptamer + linker + docking strand	GCCTGTGGTGTTGGGGCGGGTGCGTTATACATCTA
Imaging strand	CCAGATGTAT-CY3B
Thiol docking strand	2-TTATACATCTA 2 = Thiol Hexyl

Supporting Information Table 2: Resolution as determined by Fourier Ring Correlation.

Dataset	Resolution (nm) (mean ± SD)
Aggregation timecourse (0 hours)	**24.2 ± 3.95**
Aggregation timecourse (2 hours)	**24.2 ± 2.72**
Aggregation timecourse (4 hours)	**27.9 ± 5.61**
Aggregation timecourse (6 hours)	**28.8 ± 4.88**
Aggregation timecourse (8 hours)	**26.6 ± 4.77**
Aggregation timecourse (10 hours)	**26.1 ± 7.66**
Aggregation timecourse (14 hours)	**28.5 ± 5.78**
Aggregation timecourse (25 hours)	**26.5 ± 4.39**
Triplication cells (aptamer)	**36.7 ± 4.97**
Control cells (aptamer)	**48.2 ± 11.30**
Triplication cells (antibody)	**47.8 ± 16.71**
Control cells (antibody)	**41.0 ± 9.61**

Supporting Information Figures

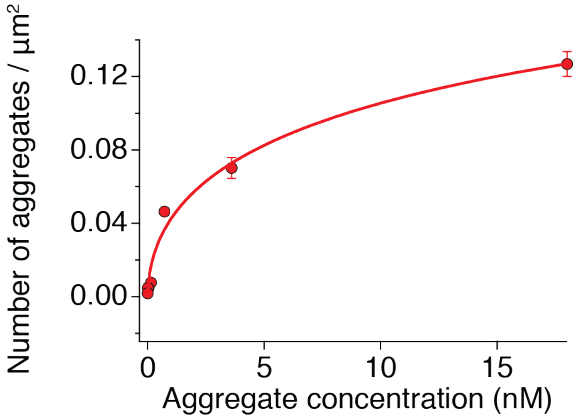

Supporting Information Figure 1: Concentration calibration curve. The number of aggregates detected with varying aggregate concentration. Points show mean ± SD (n = 3), and the data are fit to a four-parameter logistic regression[11].

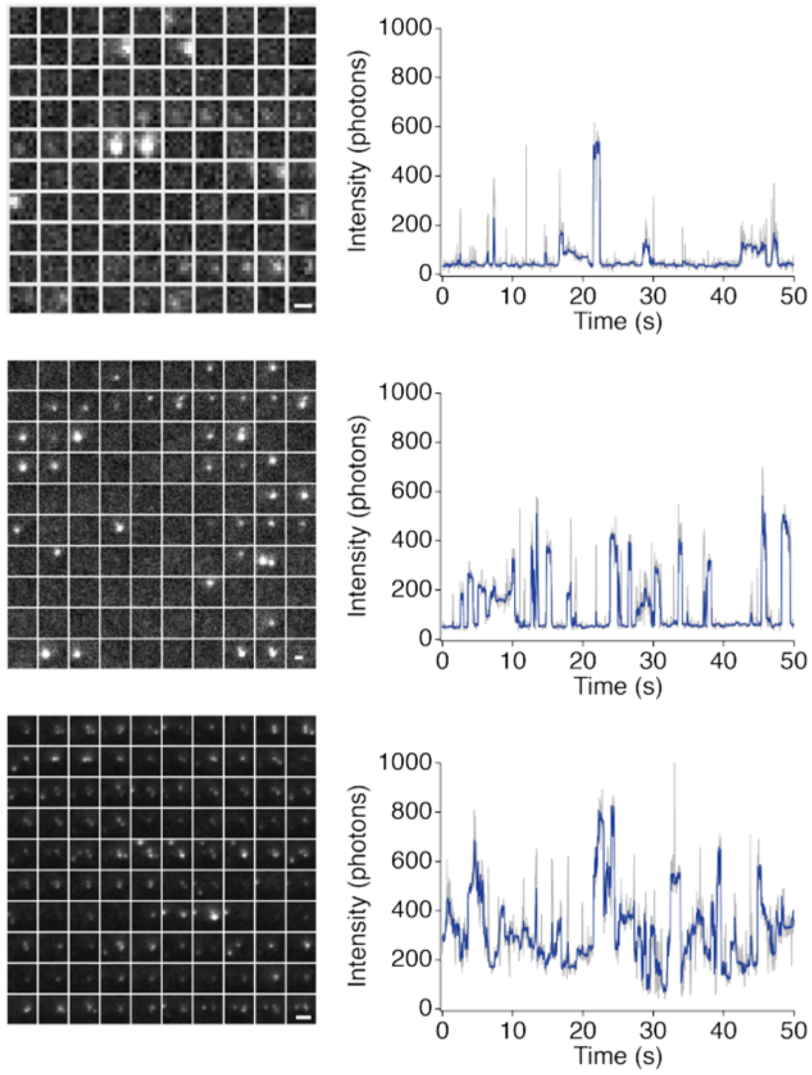

Supporting Information Figure 2: Example burst montages. Example time montages of oligomers undergoing ADPAINT. Each sub-image is separated by 0.5 s, moving through time from left to right then top to bottom, and the scale bar is 1 μm.

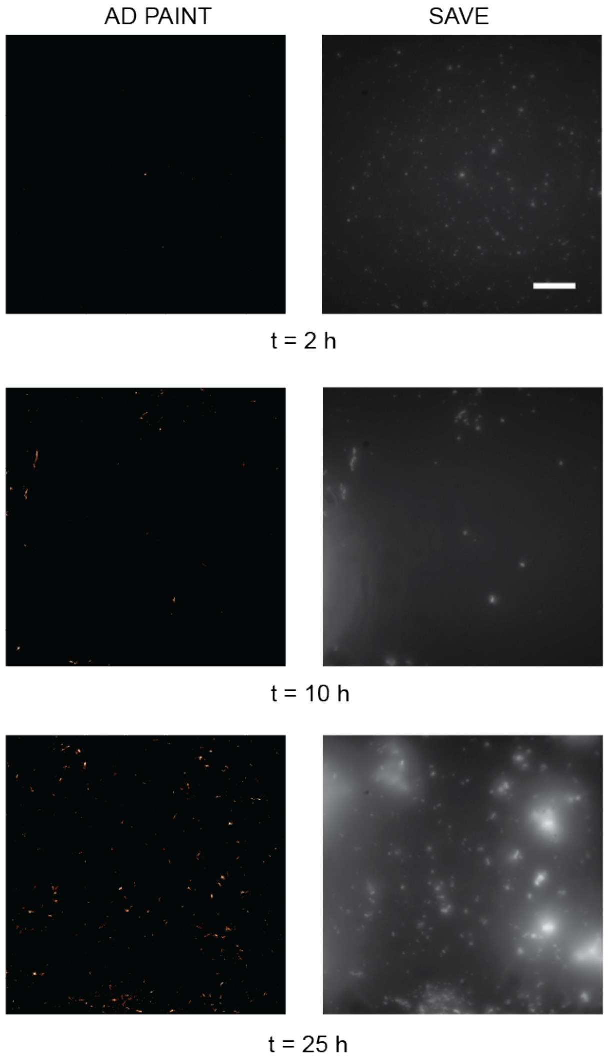

Supporting Information Figure 3: Example full field of view images of αS aggregates imaged by ADPAINT and SAVE. Three different time points are shown. (t = 2, 10, 25 h) Scale bar: 10 μm.

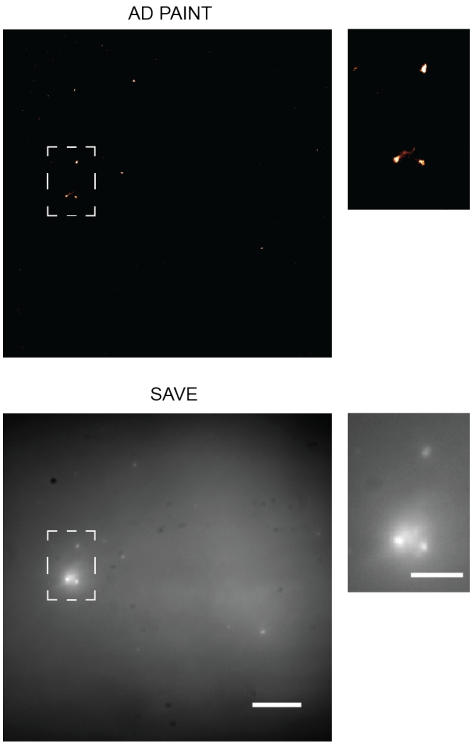

Supporting Information Figure 4: Example full field of view images of Aβ aggregates imaged by ADPAINT and SAVE. Scale bar: 10 μm (left panel), 4 μm (zoomed out right panel)

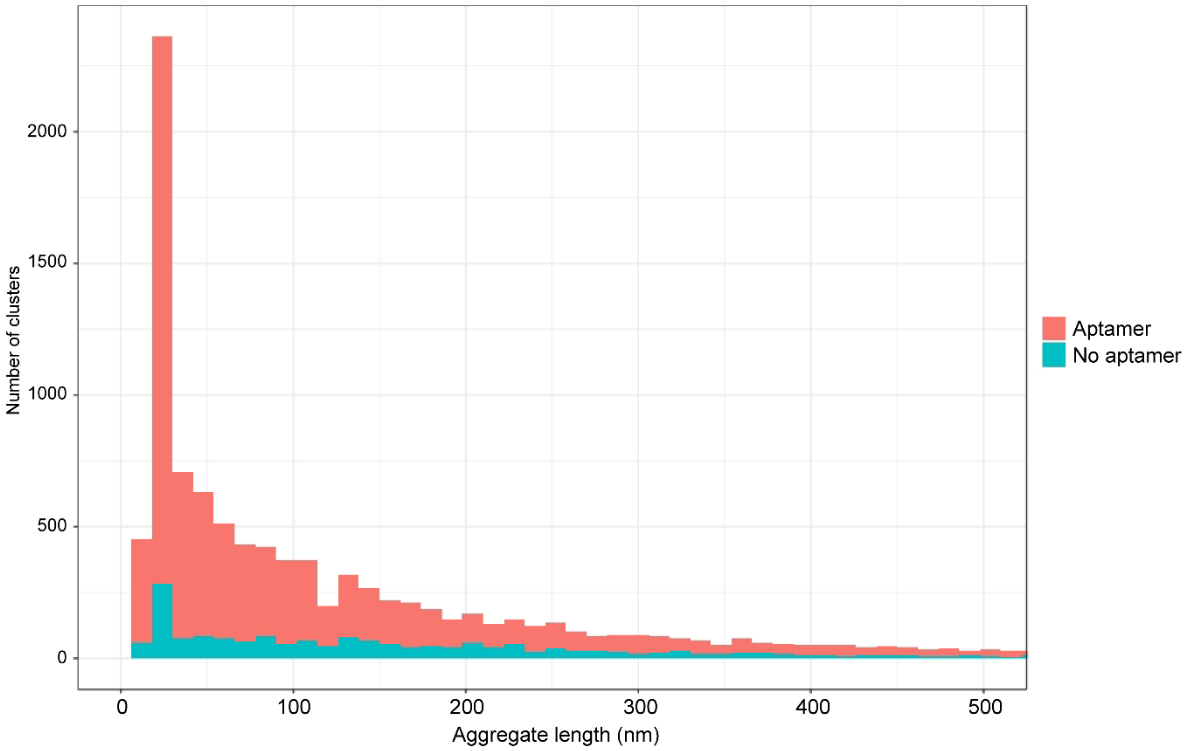

Supporting Information Figure 5: Non-specific binding of imaging strand to αS aggregates. Aggregates of αS imaged by ADPAINT were identified and sized with density-based spatial clustering of applications with noise as previously described (DBSCAN). Each cluster represents an αS aggregate from thirty-six fields of view imaged across four distinct sample wells for each experimental condition, namely the presence or absence of docking strand conjugated aptamer in the solution buffer.

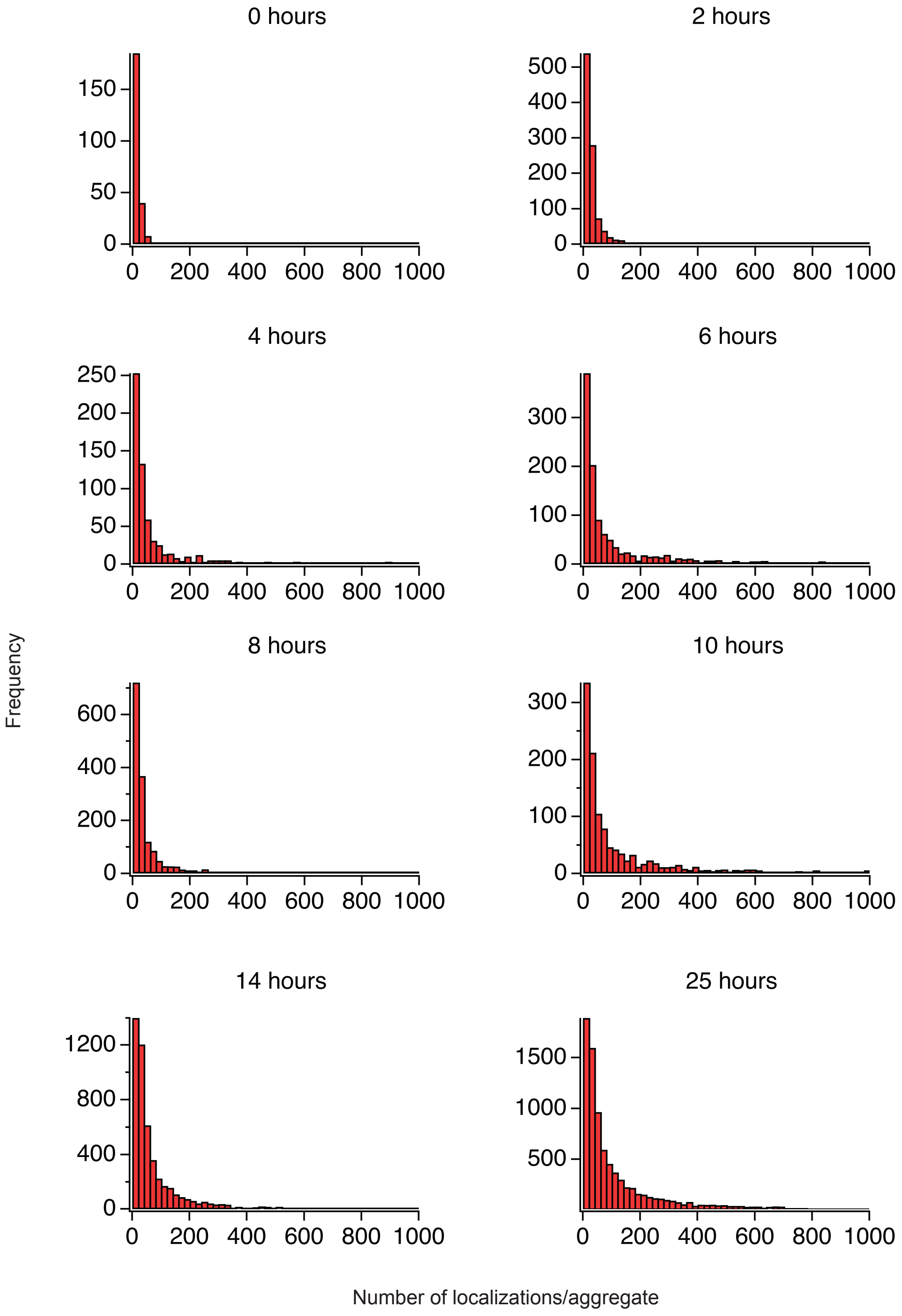

Supporting Information Figure 6: Number of localizations for each time-point. Histograms of the number of localizations for each aggregate detected over the duration of the fibril-formation process.

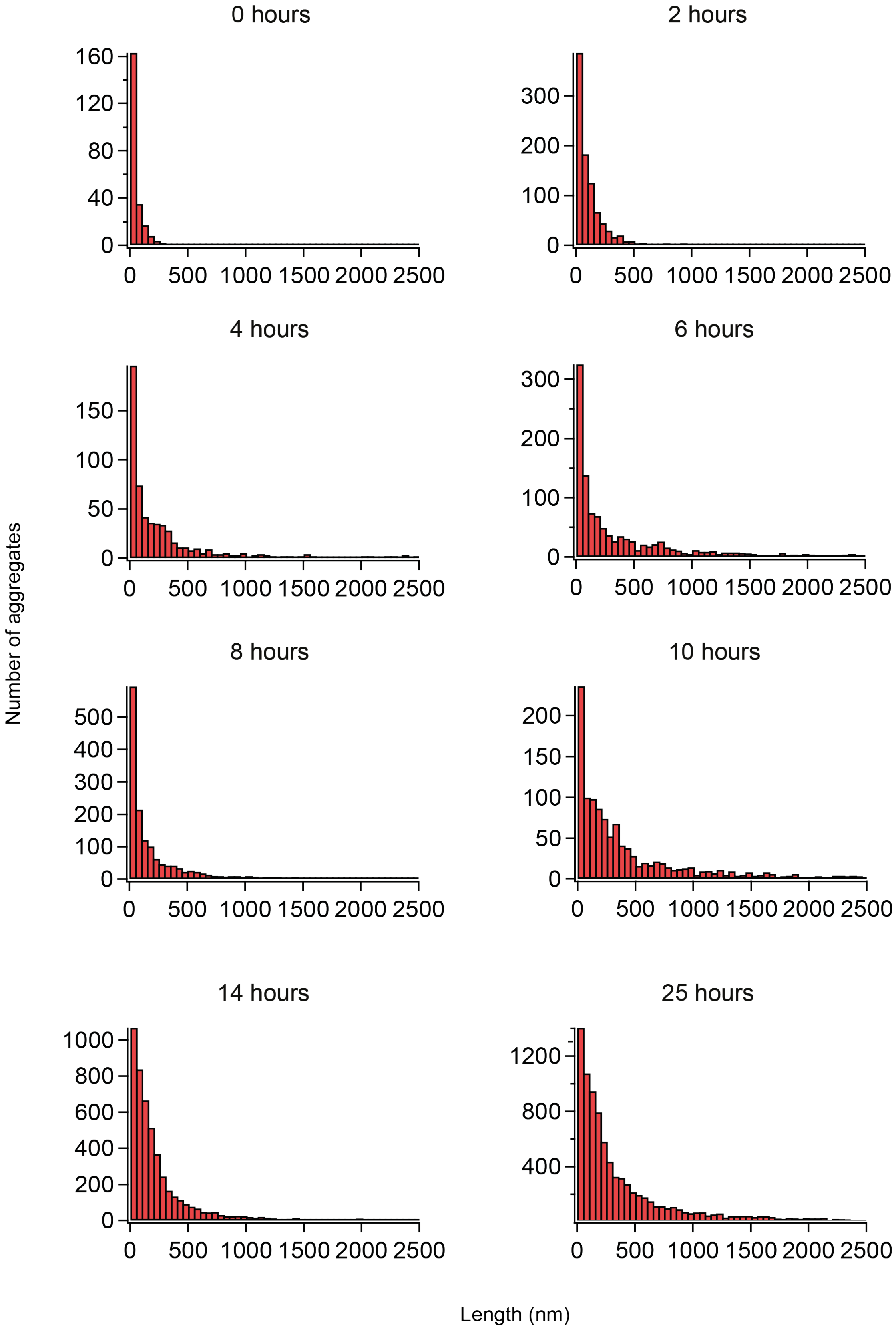

Supporting Information Figure 7: Length of aggregates for each time-point. Histograms of the length of each aggregate detected over the duration of the fibril-formation process.

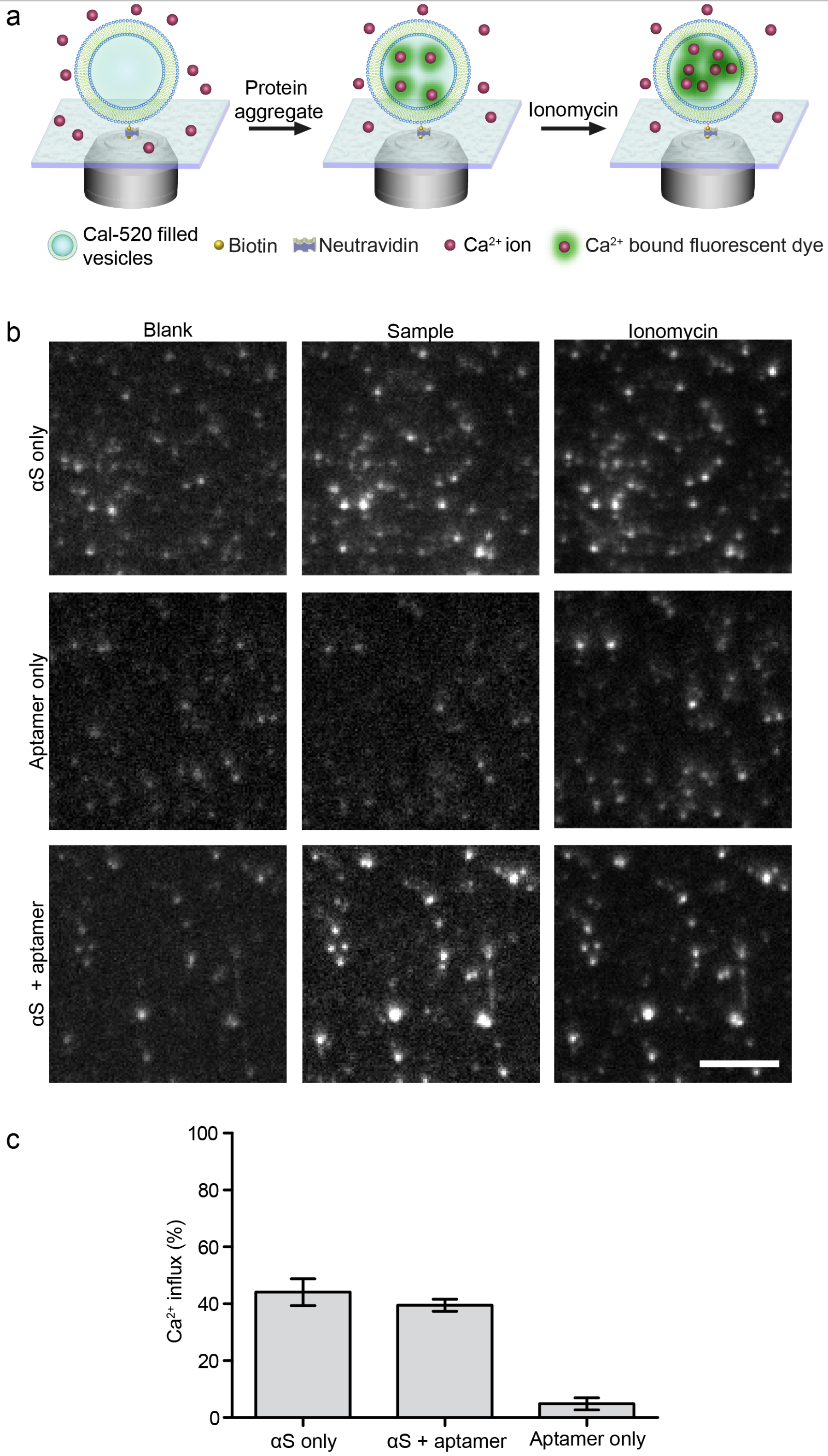

Supporting Information Figure 8: Membrane permeabilisation assay. a) Schematic representation of the membrane permeabilization assay. **b)** Example vesicles. Fluorescence is caused by Cal-520 binding Ca^{2+} that enters the vesicle when the membrane is disturbed. Scale bar is 5 µm. **c)** Quantification of membrane permeability. aS was present at 50 nM, aptamer was present at 5 µm. Values shown are means ± SD from at least 1000 vesicles.

Supporting Information Figure 11 has been uploaded as a separate file.

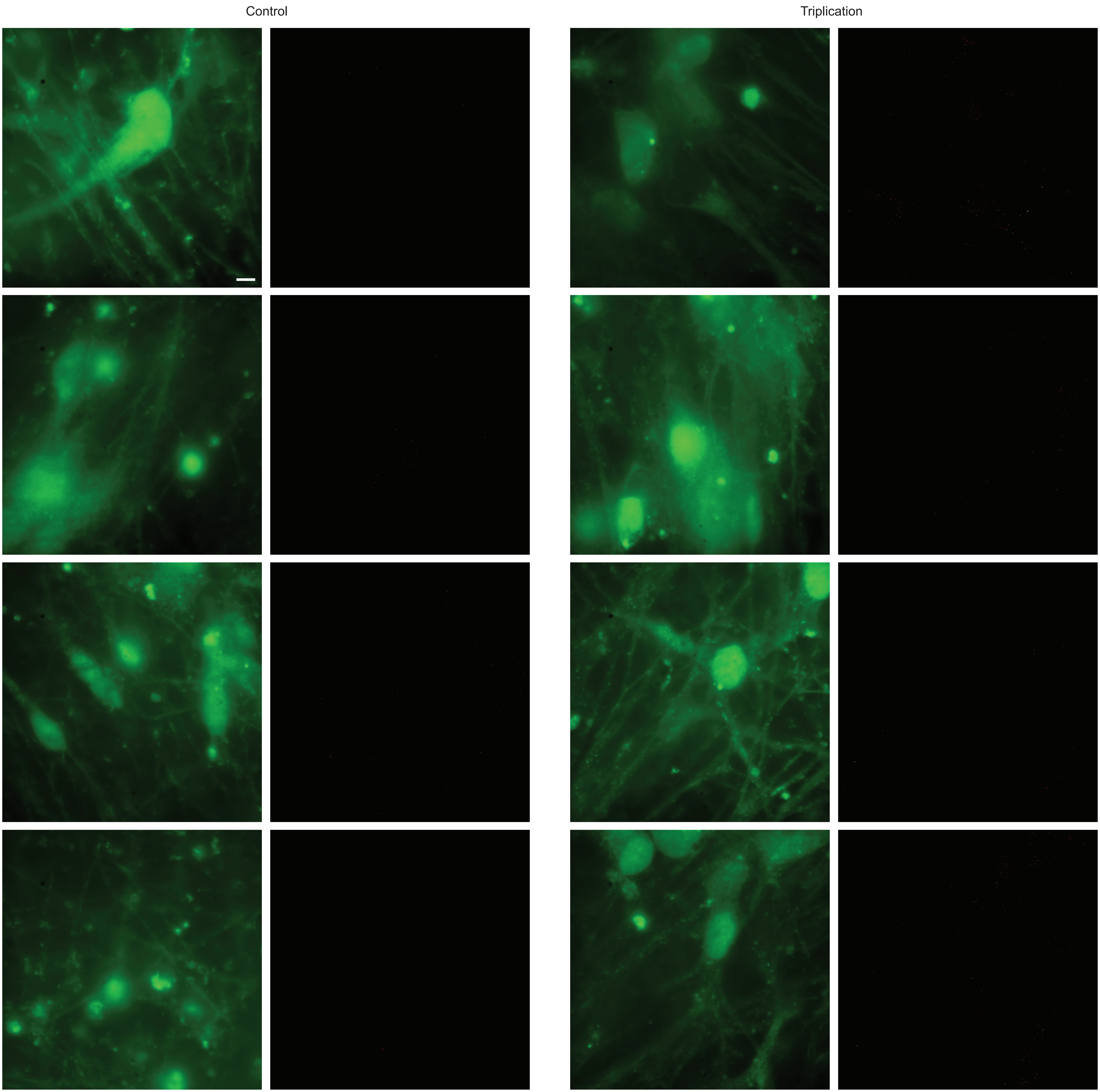
Control
Triplication

Supporting Information Figure 9: Example images from *SNCA* triplication and control iPSC-derived cortical neurons (uploaded as Supporting data). In each case, the left image (green) shows the stain with PFTAA, and the right shows the ADPAINT image. Scale bar is 5 μm.

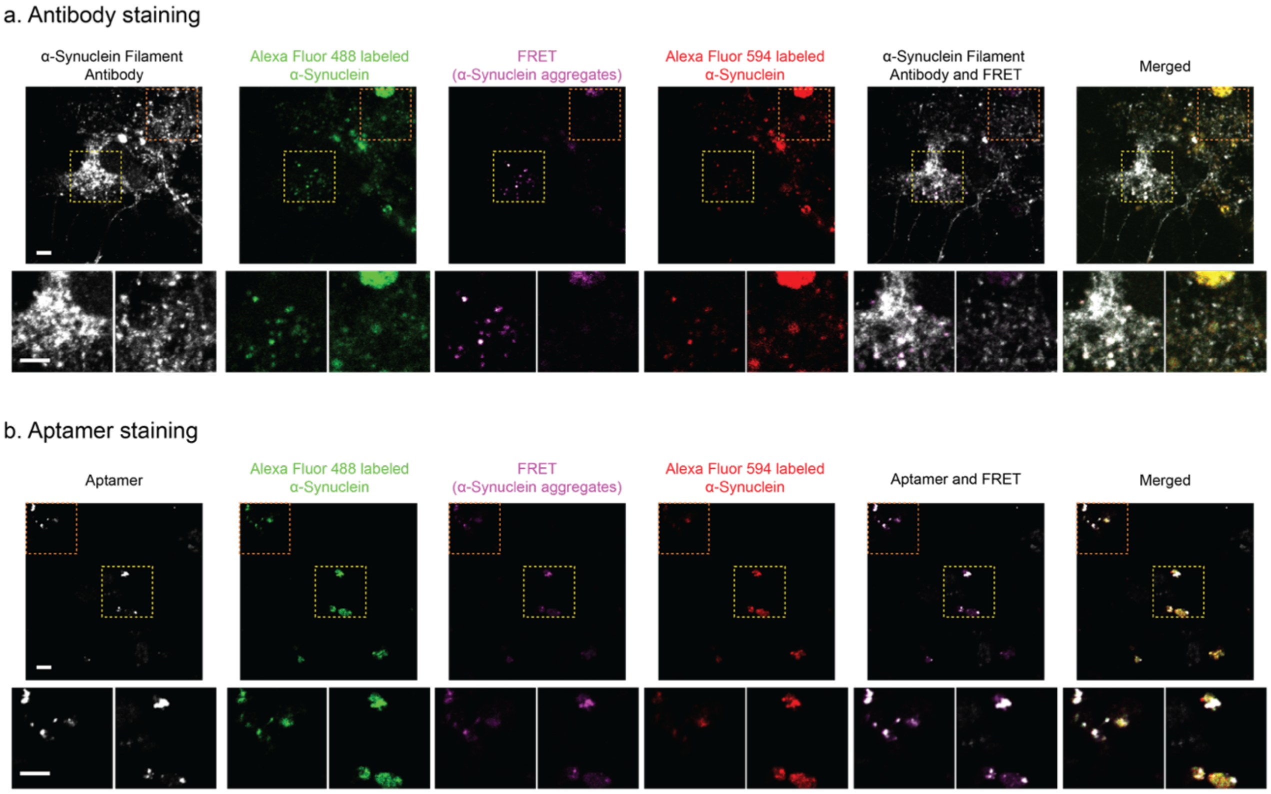

Supporting Information Figure 10: *Comparison between antibody staining and aptamer staining. αS aggregates made from an equimolar mixture of Alexa Fluor 488- and Alexa Fluor 594-labelled monomer were incubated at 70 uM for 29 h before being added at 500 nM to iPSC-derived neurons. The cells were then fixed, permeabilised and stained with either an αS filament antibody (MJF14-6-4-2) directly labelled with Alexa Fluor 647 (which should be specific to αS filaments; ab209538, Abcam) or aptamer directly labelled with Alexa Fluor 405. Aggregates taken up by the cell were detected using FRET following direct excitation at 488 nm. The antibody exhibits extensive staining throughout the cell, whereas fluorescence from the aptamer is localised to the aggregates. Scale bars are 5 μm.*

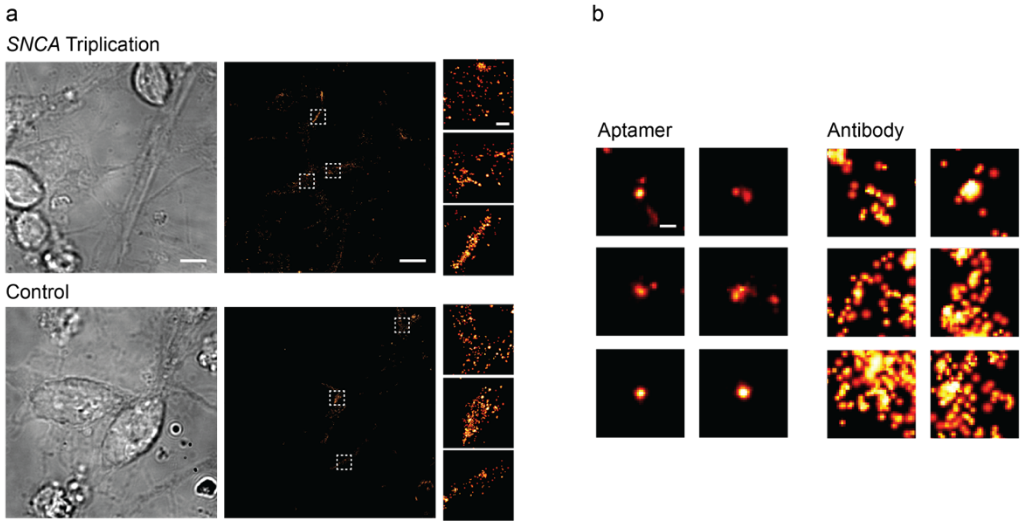

Supporting Information Figure 11: *DNA PAINT experiments with MJF14-6-4-2 antibody. (a) The MJF14-6-4-2 antibody was labelled with the docking strand before being incubated with fixed and permeabilised iPSC-derived cortical neurons overnight. Super-resolution imaging and analysis was then performed under identical conditions as for ADPAINT imaging. Unlike with ADPAINT, individual puncta*

corresponding to aggregates could not be observed, and instead staining was diffuse within the cells, which could be a result of the antibody binding monomeric αS. Furthermore, the diffuse staining could be observed in both the SNCA triplication and control cells. The scale bar is 5 µm and 500 nm in the zoom. (b) Comparison between aptamer staining and antibody staining. Individual puncta are visible with ADPAINT, whereas with the antibody, individual aggregates cannot be observed. The scale bar is 100 nm.

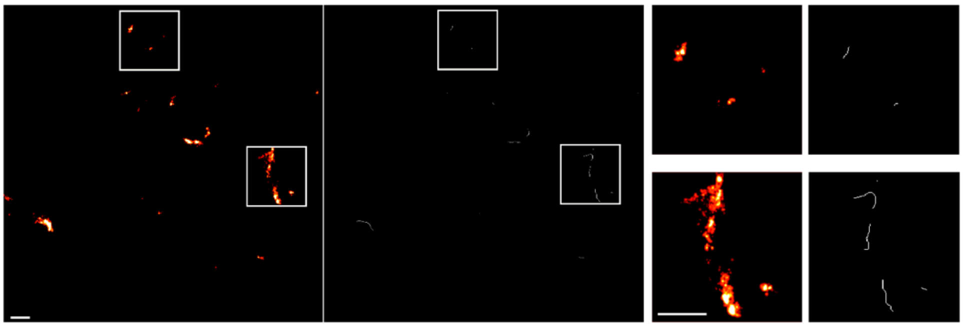

Supporting Information Figure 12: Skeletonization. Demonstration of the skeleteonization output on various sized αS aggregates. Scale bars are 1 µm.

References

[1] J. Schnitzbauer, M. T. Strauss, T. Schlichthaerle, F. Schueder, R. Jungmann, *Nat. Protoc.* **2017**, *12*, 1198–1228.
[2] M. H. Horrocks, S. F. Lee, S. Gandhi, N. K. Magdalinou, S. W. Chen, M. J. Devine, L. Tosatto, M. Kjaergaard, J. S. Beckwith, H. Zetterberg, et al., *ACS Chem. Neurosci.* **2016**, *7*, 399–406.
[3] N. Cremades, S. I. A. Cohen, E. Deas, A. Y. Abramov, A. Y. Chen, A. Orte, M. Sandal, R. W. Clarke, P. Dunne, F. A. Aprile, et al., *Cell* **2012**, *149*, 1048–1059.
[4] M. Iovino, S. Agathou, A. González-Rueda, M. Del Castillo Velasco-Herrera, B. Borroni, A. Alberici, T. Lynch, S. O'Dowd, I. Geti, D. Gaffney, et al., *Brain J. Neurol.* **2015**, *138*, 3345–3359.
[5] A. Kirkeby, S. Grealish, D. A. Wolf, J. Nelander, J. Wood, M. Lundblad, O. Lindvall, M. Parmar, *Cell Rep.* **2012**, *1*, 703–714.
[6] J. Schindelin, I. Arganda-Carreras, E. Frise, V. Kaynig, M. Longair, T. Pietzsch, S. Preibisch, C. Rueden, S. Saalfeld, B. Schmid, et al., *Nat. Methods* **2012**, *9*, 676–682.
[7] N. Banterle, K. H. Bui, E. A. Lemke, M. Beck, *J. Struct. Biol.* **2013**, *183*, 363–367.
[8] R. P. J. Nieuwenhuizen, K. A. Lidke, M. Bates, D. L. Puig, D. Grünwald, S. Stallinga, B. Rieger, *Nat. Methods* **2013**, *10*, 557–562.
[9] L. C. Serpell, J. Berriman, R. Jakes, M. Goedert, R. A. Crowther, *Proc. Natl. Acad. Sci. U. S. A.* **2000**, *97*, 4897–4902.
[10] D. A. Armbruster, T. Pry, *Clin. Biochem. Rev.* **2008**, *29*, S49–S52.
[11] J. W. A. Findlay, R. F. Dillard, *AAPS J.* **2007**, *9*, E260–E267.

Author Contributions

MHH and DK designed the study. MHH, DRW, YZ, SES performed ADPAINT experiments and analysed the data. LC, DA and MC generated the human-derived neurons. SD and PF performed the cell-permeabilization experiments. DCW, and FK provided protein aggregates for analysis. RTR provided the DNA-conjugated antibodies and assisted with experiments. SFL, CMD, SG, MGS, DK and MHH supervised the study. All authors contributed to the writing of the paper. All authors read and approved the final manuscript.
